# Modelling the Stoichiometric Regulation of C-Rich Toxins in Marine Dinoflagellates

**DOI:** 10.1371/journal.pone.0139046

**Published:** 2015-09-23

**Authors:** Adriano Pinna, Laura Pezzolesi, Rossella Pistocchi, Silvana Vanucci, Stefano Ciavatta, Luca Polimene

**Affiliations:** 1 Department of Biological, Geological and Environmental Sciences (BiGeA)—University of Bologna, Via Sant’Alberto 163, 48123 Ravenna, Italy; 2 Plymouth Marine Laboratory, Prospect Place, The Hoe, PL1 3DH Plymouth, United Kingdom; 3 Department of Biological and Environmental Sciences—University of Messina, Viale Ferdinando d’Alcontres 31, 98166 S. Agata, Messina, Italy; 4 National Centre for Earth Observation (NCEO), Plymouth Marine Laboratory, Plymouth, United Kingdom; University of Connecticut, UNITED STATES

## Abstract

Toxin production in marine microalgae was previously shown to be tightly coupled with cellular stoichiometry. The highest values of cellular toxin are in fact mainly associated with a high carbon to nutrient cellular ratio. In particular, the cellular accumulation of C-rich toxins (i.e., with C:N > 6.6) can be stimulated by both N and P deficiency. Dinoflagellates are the main producers of C-rich toxins and may represent a serious threat for human health and the marine ecosystem. As such, the development of a numerical model able to predict how toxin production is stimulated by nutrient supply/deficiency is of primary utility for both scientific and management purposes. In this work we have developed a mechanistic model describing the stoichiometric regulation of C-rich toxins in marine dinoflagellates. To this purpose, a new formulation describing toxin production and fate was embedded in the European Regional Seas Ecosystem Model (ERSEM), here simplified to describe a monospecific batch culture. Toxin production was assumed to be composed by two distinct additive terms; the first is a constant fraction of algal production and is assumed to take place at any physiological conditions. The second term is assumed to be dependent on algal biomass and to be stimulated by internal nutrient deficiency. By using these assumptions, the model reproduced the concentrations and temporal evolution of toxins observed in cultures of *Ostreopsis* cf. *ovata*, a benthic/epiphytic dinoflagellate producing C-rich toxins named ovatoxins. The analysis of simulations and their comparison with experimental data provided a conceptual model linking toxin production and nutritional status in this species. The model was also qualitatively validated by using independent literature data, and the results indicate that our formulation can be also used to simulate toxin dynamics in other dinoflagellates. Our model represents an important step towards the simulation and prediction of marine algal toxicity.

## Introduction

Blooms of toxic microalgae are increasing worldwide and their proliferation seems to be driven by several environmental and anthropogenic factors. Among these the supply of nutrients (N and P) to surface waters are of particular importance [1,2,3]. Algal toxins are highly diverse in chemical structure (ranging from N-rich alkaloids to C-based polyketides), molecular weight and mechanism of action. Van de Waal et al. [4] subdivided phytoplanktonic toxins into two main families, 1) N-rich and 2) C-rich toxins (C:N molar ratio <6.6 and >6.6, respectively), and showed that the synthesis of both groups is highly coupled to cellular stoichiometry (given as the N:P ratio). However, while N-rich toxins are stimulated only by P-limitation (and are repressed under N-limitation), C-rich toxins are stimulated by both N and P-limitation. More specifically, the content of N-rich toxins seems to be linearly correlated with the cellular N:P ratio, while for C-rich toxins the dynamics are more complicated and N:P stoichiometry alone is not sufficient for interpreting and predicting toxin dynamics [4]. This is probably due to the fact that C-rich toxins are more sensitive to several environmental factors (e.g. light, CO_2_) in addition to the limiting nutrients [2].

Under unbalanced growth conditions (e.g. under increasing C:P), the production of C-rich toxin has been interpreted as a way to store excess carbon in secondary pools [4] and/or secondary metabolites which can act as defense against grazers. The latter mechanism is also known as the carbon to nutrient balance hypothesis or CNBH [5].

Among the organisms producing C-rich toxins, *Ostreopsis* cf. *ovata* Fukuyo represents an increasing issue throughout the world, particularly in temperate areas. This microalga produces different palytoxin (PLTX)-like compounds, namely the recently re-named isobaric-PLTX (previously known as putative-PLTX) and seven analogues named ovatoxins (OVTXs): OVTX-a, -b, -c, -d/e, -f, -g [6,7,8]. When the density of this dinoflagellate in seawater is high (i.e. when it blooms) the extracellular concentration of its toxins may also reach critical levels, inducing respiratory and febrile syndrome outbreaks in humans exposed to seawater and/or marine aerosol [9,10]. These toxins may also enter the food chain and accumulate in different kinds of edible species (from mollusks to fish and crustaceans) causing severe human intoxication [11]. *O*. cf. *ovata* is known to be dangerous also for the marine environment by inducing severe mass mortalities within benthic communities [12,13,14,15].

Experimental work investigating the role of nutrients in *O*. cf. *ovata* growth and toxicity gave apparently contrasting results. Vanucci et al. [16] observed decreasing cellular growth and total toxin amount in batch cultures when shifting from balanced N:P conditions (N:P molar ratio of 16 in the culture medium, according to Redfield [17,18]), to P-deficiency (N:P = 92) and to N-deficiency (N:P = 5) conditions. By contrast, Vidyarathna & Granéli [19] observed a significantly higher toxicity (measured as hemolytic activity) in cultures grown in N-limited conditions (N:P = 1.6). Although the decrease in toxin production under nutrient depletion might represent a noteworthy exception to the CNBH, Pezzolesi and colleagues [20] observed a higher ovatoxins production concomitantly with conditions of unbalanced growth and this suggests that the stoichiometric regulation of C-rich toxin discussed in Van de Waal et al. [4] applies also to *O*.cf. *ovata*.

While the functional linkage between cellular stoichiometry and toxin production is regarded as a general feature of toxic dinoflagellates [4], a numerical model describing this function is still lacking and this limits our capacity to simulate and predict microalgal toxicity.

The objective of this paper is to develop a mechanistic model describing C-rich toxin production in marine dinoflagellates. The model was developed by interpreting the dataset presented in Pezzolesi et al. [20] describing growth and toxin production in *O*. cf. *ovata*. The choice of this organism has been driven by: 1) its ecological relevance and 2) the availability of one of the most complete datasets for a toxic dinoflagellate. The dataset used also included unpublished analyses of cellular elemental composition (C:N:P) and bacteria biomass relative to the experiments described in [20]. The new formulation has been embedded in the primary producer module of the European Regional Seas Marine Ecosystem Model (ERSEM) [21], which was simplified to reproduce a mono-specific batch culture. A sub-model describing bacterial dynamics (already present in ERSEM) was also used to mimic the bacterial activity associated with the algal cultures. The model was run by using experimental temperature and light regimes (intensity and light/dark cycle) and actual initial conditions. Model simulations provided a conceptual framework linking cellular nutrient content (given as carbon to nutrient ratio) to growth and toxin production in *O*. cf. *ovata*. Subsequently, the model was validated against independent literature data to test its applicability to different microalgae producing C-rich toxins ([4] and references therein).

## Materials and Methods

### Data

#### Experimental conditions

The numerical model was developed by interpreting the data obtained in the experiment presented in Pezzolesi et al. [20], where a strain of *O*. cf. *ovata* Fukuyo (OOAB0801) isolated in 2008 in the Western Adriatic Sea during a bloom near Bari (Italy) was used. Experimental batch cultures were prepared by adding macronutrients at a five-fold diluted f/2 concentration [22] and selenium to filtered and autoclaved natural seawater (at salinity 36). Triplicate cultures consisted of 3 L Erlenmeyer flasks, inoculated with cells collected from a culture at early stationary phase and fresh medium to a final volume of 2500 mL, maintained under illumination from cool white light at a photon flux density of 110–120 μmol m^-2^ s^-1^, at 20 ± 1°C on a 16:8 h light/dark cycle in a growth chamber.

Measurements of carbon and nitrogen in the cells were performed by filtering culture aliquots (10 mL) on GF/F glass-fiber filters (Whatman) precombusted at 550°C for 20 min. Elemental analysis was conducted using a ThermoFisher organic elemental analyzer (Flash 2000) configured for CHNS-O determination using a copper/copper oxide column. The standard 2,5-bis-(5-tert-butyl-2-benzooxazol-2-yl) thiophene (BBOT) was used for calibration. Particulate phosphate was measured photometrically (UV/VIS, JASCO 7800, Tokyo, Japan) after digestion with a solution of 5% potassium peroxide using the method described by [23].

#### Algal and bacteria carbon

**Fig 1:**
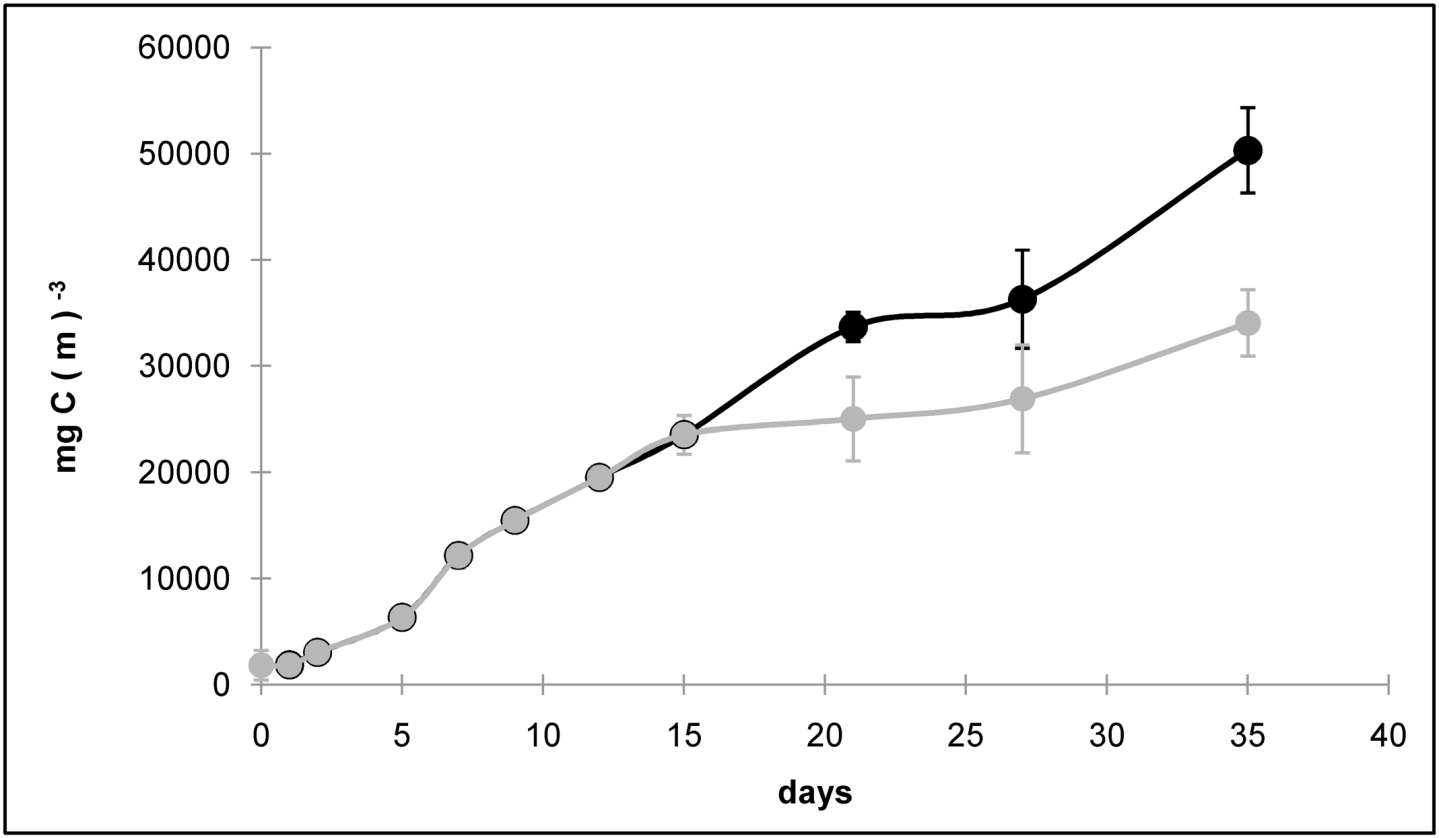
Growth curve of *O*. cf. *ovata* observed in the cultures. Black spots represent the POC biomass originally detected by CHN analyzer, while the grey ones are the values corrected through regression.

A growth-curve describing the evolution of carbon biomass of *O*. cf *Ovata*, was built by using data from CHN analyses (Fig 1). However, it is likely that the particulate organic carbon (POC) detected by the CHN was affected by the presence of the extra-cellular polysaccharidic mucilage, which is copiously produced by *O*. cf. *ovata* particularly during the stationary phase [24,19,20,25]. This has probably generated the extremely high POC to PON and POC to POP ratios measured at the end of the experiment (up to 25 and 500 for molar C:N and C:P ratios, respectively). The latter values are extremely high if compared to both field [26,27] and laboratory [28,29,30] observations for marine microalgae. To overcome this problem, the last part of the POC curve was corrected by using the regression line describing the correlation (r>0.99; p<0.01, see S1 Fig) between the POC and the total cell volume in the first 15 day of the experiment, when the presence of mucilage was low and so POC was a good estimator of algal carbon. The presence of bacteria and their growth in the algal cultures were assessed by direct bacteria counts using epifluorescence microscopy after staining with SYBR gold [31].

Bacteria biomass was estimated from cell counts and mean cell volumes assuming a carbon content of 145 fg C μm^-3^ [32,33]. Bacterial nitrogen and phosphorus were calculated assuming a fixed C:N:P molar ratio of 45:9:1 [34].

#### Toxins

**Fig 2:**
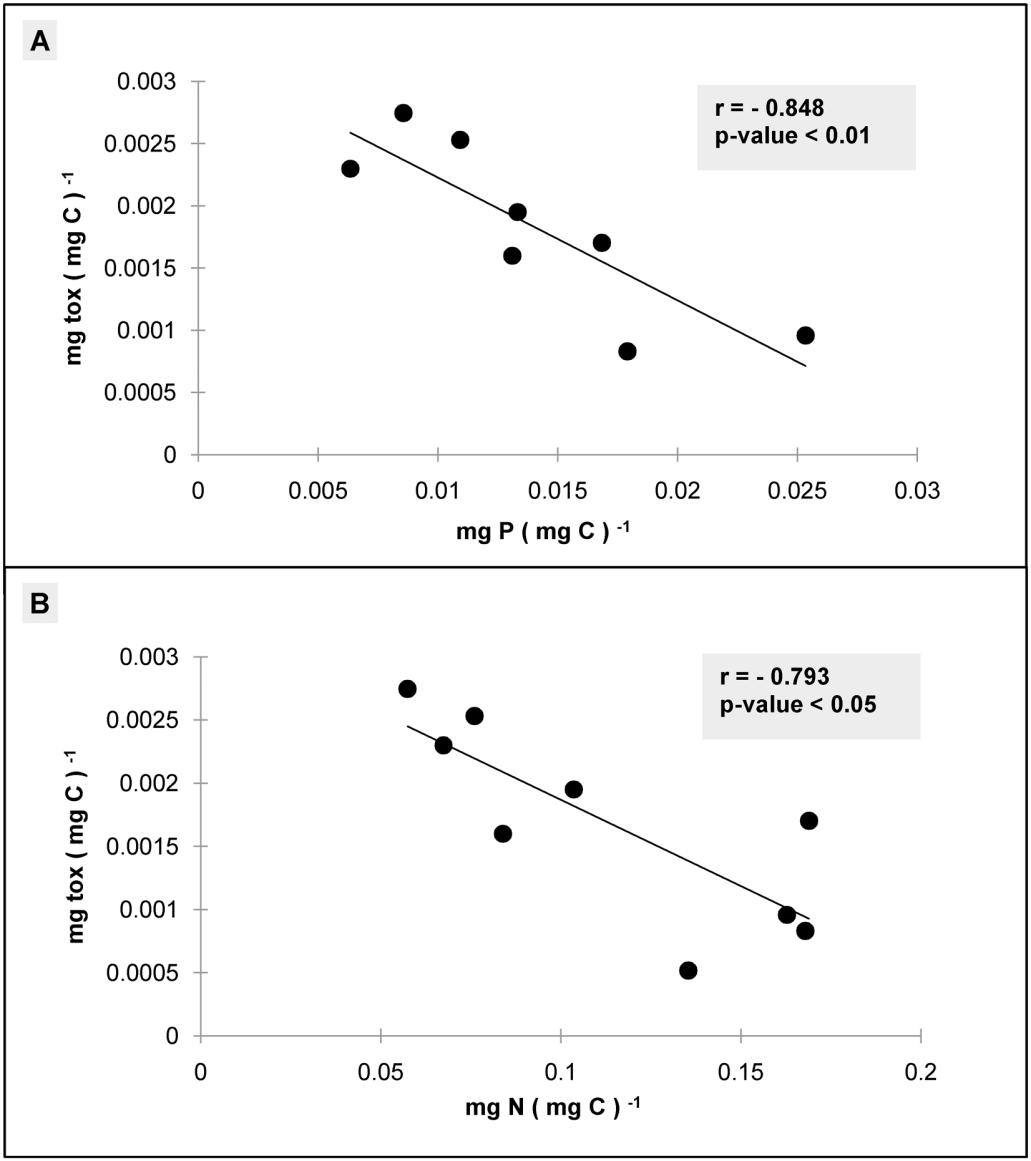
Relationship between toxin to carbon ratio and phosphorus (A) and nitrogen (B) to carbon ratios observed in the experiment presented in Pezzolesi et al. [20].

Toxins produced in the cultures were treated as a single “generic” toxic compound with elemental formula C_129.66_ H_224.32_ N_3_ O_52.53_. This generalization (which was necessary for model comparison, see next section) is plausible considering that the observed toxins (isobaric-palytoxin, ovatoxin-a, -b, -c, -d, -e) were similar in terms of both molecular structure and stoichiometry (129–131 atoms of C, 223–227 atoms of H, 3 atoms of N, 52–54 atoms of O) and that their relative proportions were stable throughout the experiment as previously observed [6,35]. The relationship observed between toxin cellular content (given as toxin to carbon ratio) and cellular nutritional status (given as carbon to nutrients ratios) is displayed in Fig 2.

### The model

Model equations (largely derived from Blackford et al. [21]) are given in Appendix A (S1 File). Here we present the equations of toxin dynamics and the ERSEM equations which have been modified with respect to Blackford et al. [21]. Model parameters are listed in Table 1.

**Table 1:** Model parameters.

Parameter description	Notation	Unit	Value	Reference
***Optical and photosynthetic parameters***				
Background extinction	*σ*_*bg*_	m^-1^	0.06	This work
Extinction coefficient of semi-labile DOM	*σ*_*sl*_	m^2^ (mg C)^-1^	1 E-5	This work
Extin. coeff. of POM	*σ*_*POM*_	m^2^ (mg C)^-1^	1 E-4	[21]
Extin. coeff. of algal biomass	*σ*_*P*_	m^2^ (mg C)^-1^	4 E-4	[21]
Max. spec. photosynthetic rate	*r*_*ass*_	d^-1^	1.5	[21]
Max. Chl/C cell ratio	*θ*_*max*_	mg Chl (mg C)^-1^	0.02	[37]
Initial slope of PI-curve	*α*	mg C m^2^ (mg Chl W d)^-1^	2.5	This work
Photo-inhibition parameter	*β*	mg C m^2^ (mg Chl W d)^-1^	0.04	This work
***Other algal parameters***				
Q_10_ parameter	*Q*_10_	-	2	[21]
Basal respiration rate	*r*_*B*.*res*_	d^-1^	0.01	This work
Minimum lysis rate	*r*_*lys*_	d^-1^	0.005	This work
Respired fraction of PP	*r*_*A*.*res*_	-	0.25	[21]
Fraction of excreted carbon	*p*_*A*.*exu*_	-	0.2	This work
Minimum N/C ratio	*Q*_*Nmin*_	mmol N (mg C)^-1^	0.003	This work
Redfield N/C ratio	*Q*_*NRed*_	mmol N (mg C)^-1^	7.862 E-4	[17]
Minimum P/C ratio	*Q*_*Pmin*_	mmol P (mg C)^-1^	1.5 E-4	This work
Redfield P/C ratio	*Q*_*PRed*_	mmol P (mg C)^-1^	1.26 E-2	[17]
Min. N/C ratio for Chl synthesis	$Q_{Nmin}^{chl}$	mmol N (mg C)^-1^	0.00687	This work
Min. P/C ratio for Chl. Synthesis	$Q_{Pmin}^{chl}$	mmol P (mg C)^-1^	4.288 E-4	This work
Affinity for NO_3_^-^	*a*_*N3*_	m^3^ (mg C)^-1^ d^-1^	0.01	This work
Affinity for NH_4_^+^	*a*_*N4*_	m^3^ (mg C)^-1^ d^-1^	0.01	This work
Affinity for PO_4_^3-^	*a*_*p*_	m^3^ (mg C)^-1^ d^-1^	0.01	This work
N-stress threshold	*S*_*N*_	-	0.6	This work
P-stress threshold	*S*_*P*_	-	0.6	This work
N-threshold for max. uptake	$s_{N}^{upt}$	-	0.95	This work
P-threshold for max. uptake	$s_{P}^{upt}$	-	1.2	This work
N-stress threshold for chlorophyll synthesis	$s_{N}^{chl}$	-	0.95	This work
P-stress threshold for chlorophyll synthesis	$s_{P}^{chl}$	-	1.2	This work
N-stress threshold for respiration decrease	$s_{N}^{res}$	-	0.56	This work
P-stress threshold for respiration decrease	$s_{P}^{res}$	-	0.51	This work
Carbon fraction invested in basal toxin production	*Q*_*Tmin*_	-	0.001	This work
Maximum tox:C ratio	*Q*_*Tmax*_	-	0.003	This work
Carbon fraction invested in toxin production under nutrient stress	*φ*	d^-1^	0.0025	This work
***Bacterial and organic matter parameters***				
Q_10_ parameter for bacteria	*Q*_10*B*_	-	2	[21]
Max spec. uptake rate	$r_{ass}^{B}$	d^-1^	4	[21]
Respired fraction of uptake	$r_{A.res}^{B}$	-	0.6	This work
Respired fraction of uptake under low oxygen	$r_{resox}^{B}$	-	0.2	[21]
Basal respiration at 10°C	$r_{B.res}^{B}$	d^-1^	0.05	This work
Mortality rate	$r_{lys}^{B}$	d^-1^	0.05	[21]
Half saturation constant for oxygen limitation	*h*_*ox*_	-	0.312	[21]
Maximum N/C ratio	$Q_{Nmax}^{B}$	mmol N (mg C)^-1^	0.017	[39]
Maximum P/C ratio	$Q_{Pmax}^{B}$	mmol P (mg C)^-1^	0.0018	[39]
Half saturation constant for NH_4_ uptake	*h*_*N*_	mmol N m^-3^	0.5	[21]
Half saturation constant for PO_4_ uptake	*h*_*p*_	mmol P m^-3^	0.2	This work
Fraction of labile DOM derived from lysis and exudation	*r*_*detr*_	-	0.25	This work
Breakdown of semi-labile to labile DOM	*r*_*dis*_	d^-1^	0.01	This work
Toxin degradation rate	*t*_*deg*_	d^-1^	0.1	This work

#### Toxin production and fate

Toxic compounds were modelled through two distinct state variables: *tox*, indicating cellular toxins, and *toxe*, indicating extra-cellular toxins. Production of cellular toxin was assumed to be composed of two additive terms. The first term accounts for a constant (moderate) production assumed to be dependent on the newly photosynthesized carbon and takes place under any nutrient conditions. The second term depends on biomass and it is only active under intra-cellular nutrient limiting conditions, represented as internal nutrient to carbon ratio:

$$\frac{\partial tox}{\partial t}{|}^{INC}\;=\;{\left[\left(GPP-\;A.RES-EXU\right)\cdot Q_{Tmin}\right]}_{basal}+{\left[\left(1-NS\right)\;\cdot \left(1-\frac{Q_{T}}{Q_{Tmax}}\right)\cdot P^{C}\cdot \varphi \right]}_{stress}$$ (1)

where *GPP* is the gross primary production, *A*.*RES* is the activity respiration and *EXU* is the “physiological” exudation associated with the gross primary production. *Q*_*Tmin*_, *Q*_*T*_, and *Q*_*Tmax*_ are the minimum, actual and maximum toxin to carbon ratio, respectively; *P* ^*C*^ is the algal biomass; *φ* is the fraction of cellular carbon daily invested in the production of *tox* during nutrient-stress conditions. *NS* is the function describing nutrient limitation and is given by:

$$NS=MIN\left(NS_{P},NS_{N}\right)$$ (2)

*NS*_*i*_(*i* = N, P) is given by:

$$NS_{i}=MIN\;\left\{1\;,\;MAX\;\left[0\;,\;\frac{Q_{i}-Q_{i\;min}}{\left(s_{i}\cdot Q_{i\;Red}\right)-Q_{i\;min}}\right]\right\}$$ (3)

where *Q*_*i*_ (*i* = N, P) are the actual nutrient to carbon ratios and *Q*_*i min*_ are the minimum nutrient to carbon ratios, respectively [36]. *S*_*i*_ (*i* = N, P) are threshold-parameters which determine the nutrient to carbon ratios, given as fraction of the Redfield ratios [18], that enhance the cell production of toxin.

It should be stressed that both *tox* and *toxe* are modelled through their carbon content. This implies that toxin nitrogen content is assumed negligible with respect to the total cellular nitrogen budget. Toxin loss terms were assumed to be composed of lysis, basal respiration and nutrient stress-driven exudation:

$$\frac{\partial \;tox}{\partial \;t}{|}^{LOSS}=\;\left(r_{lys}+\frac{B.RES}{P^{C}}+\frac{S.EXU}{P^{C}}\right)\cdot tox$$ (4)

where *r*_*lys*_ is the lysis rate, *B*.*RES* is the basal respiration (Eq 10) and *S*.*EXU* is the nutrient stress-driven exudation [21] (Eq 10a in S1 File).

The amount of toxins released outside the cell is channeled into *toxe*:

$$\frac{\partial \;toxe}{\partial \;t}{|}^{INC}=\left(r_{lys}+\frac{S.EXU}{P^{C}}\right)\cdot tox$$ (5)

We assumed that released toxins become part of the dissolved organic pool and are degraded by bacteria with a temporal delay:

$$\frac{\partial \;toxe}{\partial \;t}{|}^{loss}=UPTAKE_{B}\cdot t_{deg}\cdot \frac{toxe}{DOC}$$ (6)

where *UPTAKE*_*B*_ is the bacteria uptake of DOC (see S1 File), *t*_*deg*_ is the toxin degradation rate (see Table 1) and *DOC* is the concentration of the labile dissolved organic carbon.

#### Chlorophyll, basal respiration and mortality

We modified the chlorophyll dynamics described in Blackford et al. [21] to reproduce better the observed temporal evolution of chlorophyll and the chlorophyll to carbon ratio. The dependence of chlorophyll production on nutrient stress was modelled by introducing a new factor *NS* ^*chl*^ accounting for nutrient limitation in the source equation of chlorophyll synthesis:

$$\frac{\partial \;chl}{\partial \;t}{|}^{INC}=\left(PHOTOSYNTHESIS-\;A.RES\right)\cdot \rho \cdot NS^{chl}$$ (7)

where *PHOTOSYNTHESIS* is the gross primary production (Eq 5a in S1 File) and *A*.*RES* is the activity respiration (Eq 12a in S1 File). *ρ* is the variable fraction of freshly synthesized carbon invested in chlorophyll synthesis [37] (see Eq 7a in S1 File). *NS* ^*chl*^ is given by:

$$NS^{chl}=MIN\left(NS_{P}^{chl}\;,\;NS_{N}^{chl}\right)$$ (8)

${NS}_{i}^{chl}$(*i* = N, P) is given by:

$$NS_{i}^{chl}\;=MIN\left\{1\;,\;MAX\left[0\;,\;\frac{Q_{i}-Q_{i\;min}^{chl}}{\left(s_{i}^{chl}\cdot Q_{i\;Red}\right)-Q_{i\;min}^{chl}}\right]\right\}$$ (9)

where $Q_{i\;min}^{chl}$ (*i* = N, P) are the minimum nutrient to carbon ratios for chlorophyll synthesis and $\;s_{i}^{chl}$ are factors that determine the fraction values of the Redfield nutrient to carbon ratios at which the chlorophyll synthesis starts to decrease.

We re-formulated the ERSEM equations describing lysis and basal respiration in order to reproduce the biomass concentration observed in the last part of the experiment. In comparison to Blackford et al. [21] the lysis rate was reduced (see Table 1) and assumed to be independent of nutrient stress (Eq 8a in S1 File). This choice was driven by the observation of a very small number of dead cells in the cultures, which suggested that *O*. cf. *ovata* was only marginally affected by lysis in the investigated system.

A reduction of basal respiration under extreme nutrient limiting conditions was assumed, to mimic the formation of resting stages [38]:

$$B.RES={ʄ}^{T}\cdot r_{R.res}\cdot NS^{res}\cdot P^{C}$$ (10)

where ʄ^*T*^ is the function describing the metabolic dependency from temperature [21] (Eq 3a in S1 File) and r_B.res_ is the basal respiration rate. The value of the limiting factor NS^*res*^ varies between 0.1 and 1, and is given by:

$$NS^{res}=MIN\;\left\{1\;,\;MAX\;\left[0.1\;,\;MIN\;\left(NS_{P}^{res}\;,\;NS_{N}^{res}\right)\right]\right\}$$ (11)

where ${NS}_{i}^{res}$(*i* = N, P) is given by:

$$NS_{i}^{res}=\frac{Q_{i}-Q_{i\;min}}{\left(s_{i}^{res}\cdot Q_{i\;Red}\right)-Q_{i\;min}}$$ (12)

where $s_{i}^{res}$ are multiplying factors which describe the fraction values of the Redfield nutrient to carbon ratios at which the basal respiration rate starts to decrease.

Finally, given the high concentration of dissolved polysaccharides observed in the cultures, we assumed that the semi-labile dissolved organic carbon ($R_{sl}^{C}$) excreted by the alga was able to reduce the available light, following the equation describing light extinction reported in Appendix A (Eq 1a in S1 File).

### Sensitivity analysis with respect to selected processes

A process-based sensitivity analysis was performed by modifying the formulation described above and assessing the consequent changes in model outputs. More specifically, three simulations S1-S3 were carried out by “switching off” three key processes directly linked to our model assumptions: S1) the reduction of basal respiration under extreme nutrient limiting conditions (Eq 10); S2) the nutrient stress-induced reduction of chlorophyll synthesis (Eqs 7 and 8) and S3) the enhanced production of toxin triggered by nutrient stress (Eq 1). An additional simulation was carried out by removing the bacteria component from the model. Results were compared with the reference simulation and with the experimental data in order to quantify to what extent the above mentioned processes and the presence of bacteria affected the simulation of *O*. cf. *ovata* growth and in particular toxin dynamics.

### Sensitivity analysis with respect to model parameters

A quantitative sensitivity analysis was carried out to rank the importance of the model parameters in determining the model output. We applied a Monte-Carlo based approach (e.g. [40,41]) to rank the sensitivity of the simulated maximum value of the concentration of intracellular toxin with respect to the model parameters listed in Table 1. The m model parameters X_j_ are collected in the “input factor” vector, **X**_**i**_ = (X_1_,..,X_j_, …,X_m_), i = 1,2,…,n. A number n = 1000 random realizations of the vector was obtained by sampling uniform probability distributions defined for each of the parameters. The range of such distributions was set equal to ±30% of the reference values of the parameters listed in Table 1. The 30% variation with respect to the reference values of the parameters is often assumed in sensitivity analyses of environmental models when the real ranges are unknown (see, e.g., Ciavatta et al. [42]). We considered the 30% range to be reasonable also for the parameters of our model. Each random realization was used to run a model simulation that provided a scalar output y_i_. The parameter-to-output relationship was represented by means of a multiple linear regression model **y** = **X b +ε**. The absolute values of the standardized regression coefficients, |β_j_|, are the sensitivity indexes that provide the rank of the input factors (e.g., [41,43]). We note that the regression coefficients provide meaningful rankings of the parameters only when the linear regression explains a relatively large fraction of the variability of the model output [43]. We assessed the applicability of the linear regression by computing the fraction of explained variance (R^2^), the regression significance (F-statistic of the null hypothesis of constant model, p<0.01), as well as the significance of the standardized regression coefficients (t-statistic, p<0.05).

### Comparison with literature data

Literature on C-rich toxin producers was reviewed and datasets suitable for model validation were selected. The observational values used for model comparison were obtained by using data describing the carbon to phosphorous ratios (C:P) and the carbon associated with toxin as a percentage of total carbon (C-tox:C)×100 observed in different strains of *Karenia brevis* [44,45]. Data related to N-limitation were taken from table 2 (C:N) and from Figs 1F and 2F [(C-tox:C) ×100] of Hardison et al. [44]. Data related to P-limitation were taken from Table 3 of Hardison et al. [45]. The increase of cellular toxin for each strain considered was obtained by dividing the values of (C-tox:C) ×100 observed under nutrient depletion conditions by the same values observed under nutrient replete conditions. The increments of toxin were then averaged (and the standard deviation was computed) over all the strains investigated. Overall, we used 10 values of C:P and (C-tox:C) ×100 (each of them given as average of three replicates, [45]) to assess the model performance under P-limitation and 4 values of C:N and (C-tox:C) ×100 (each of them given as average of three replicates, Hardison et al. [44]) to assess the model performance under N-limitation. For this comparison, the model was run with the initial phosphate and nitrate concentrations reported in Hardison et al. [44,45] and without bacteria component. We also assumed balanced initial conditions for chlorophyll and internal toxin. The simulated toxin to carbon ratios were normalized by the value assumed to represent the toxin to carbon ratio under balanced growth (0.001, see Table 1 and Eq 1).

## Results

### Model simulations and comparison with experimental data

**Fig 3:**
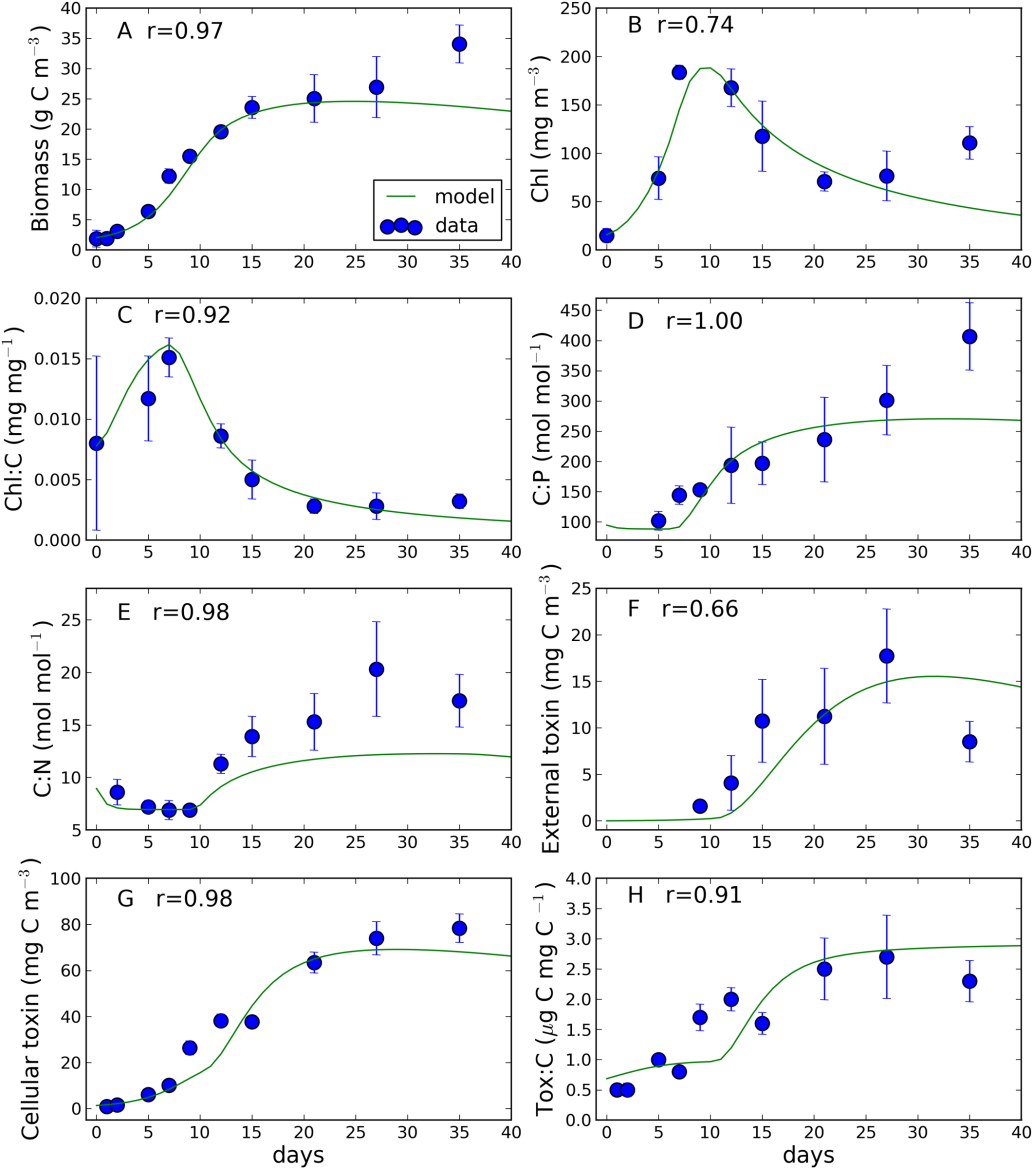
Comparison between observed and simulated (A) algal biomass (Biomass), (B) chlorophyll (Chl), (C) chlorophyll to carbon ratio (Chl:C), (D) carbon to phosphorus ratio (C:P), (E) carbon to nitrogen ratio (C:N), (F) external toxin, (G) cellular toxin and (H) toxin to carbon ratio (Tox:C). Bars indicate standard deviations. In each panel the Spearman rank correlation coefficient (r) between observations and simulations is also displayed. All the correlations are significant (p<0.05) except for panel F.

The comparison between model simulation and experimental data is shown in Fig 3. All the model outputs were in good agreement with the experimental data, as highlighted by the high values of the correlation coefficient reported in the figure.

The simulated evolution of the algal biomass (Fig 3A) depicted a “classic” growth curve. After a first phase of acclimation (days 0–3), biomass started to increase exponentially (days 3–9) reaching a stable value (~25 g C m^-3^) after 15 days (stationary phase). The model simulation reproduced the observed pattern well, with the exception of the last value (day 35), which was underestimated by the model. Simulated chlorophyll concentration (panel B) peaked on day 8, reaching 185 mg m^-3^. Afterwards, chlorophyll decreased monotonically down to 42 mg m^-3^ at day 35. The model reproduced well the observed pattern of chlorophyll until day 21. However, the observed chlorophyll increased again in the last two sampling days (27 and 35), reaching a value of ~100 mg m^-3^ at the end of the experiment. Simulated chlorophyll to carbon ratio (Chl:C, panel C) displayed a behavior similar to the modelled chlorophyll and is in good agreement with the observations. Carbon to nutrient ratios (panels D and E) simulated by the model presented a sharp increase from day 5 (phosphorus) and 9 (nitrogen). The carbon to nitrogen ratio stabilized after day 21 (reaching a value of 12 mol mol^-1^) while the carbon to phosphorus ratio increased until the end of the experiment (when it reached a value of ~270 mol mol^-1^). Model simulations reproduced well the observed dynamics although underestimating the carbon to nitrogen ratio in the second half of the experiment.

External toxin concentration (panel F) was detectable only after 9 days and reached its maximum value (~15 mg m^-3^) at day 27 while it decreased to 6 mg C m^-3^ at the end of the experiment. Model simulation was in qualitatively good agreement in terms of both magnitude and qualitative behavior until day 28, but it overestimated the observed values in the last part of the experiment (15 mg m^-3^ simulated against 8 mg m^-3^ observed). The correlation between observed and simulated external toxin was the only one that was not significant (p = 0.07), probably as a consequence of the relatively small number (6) of available data points.

**Fig 4:**
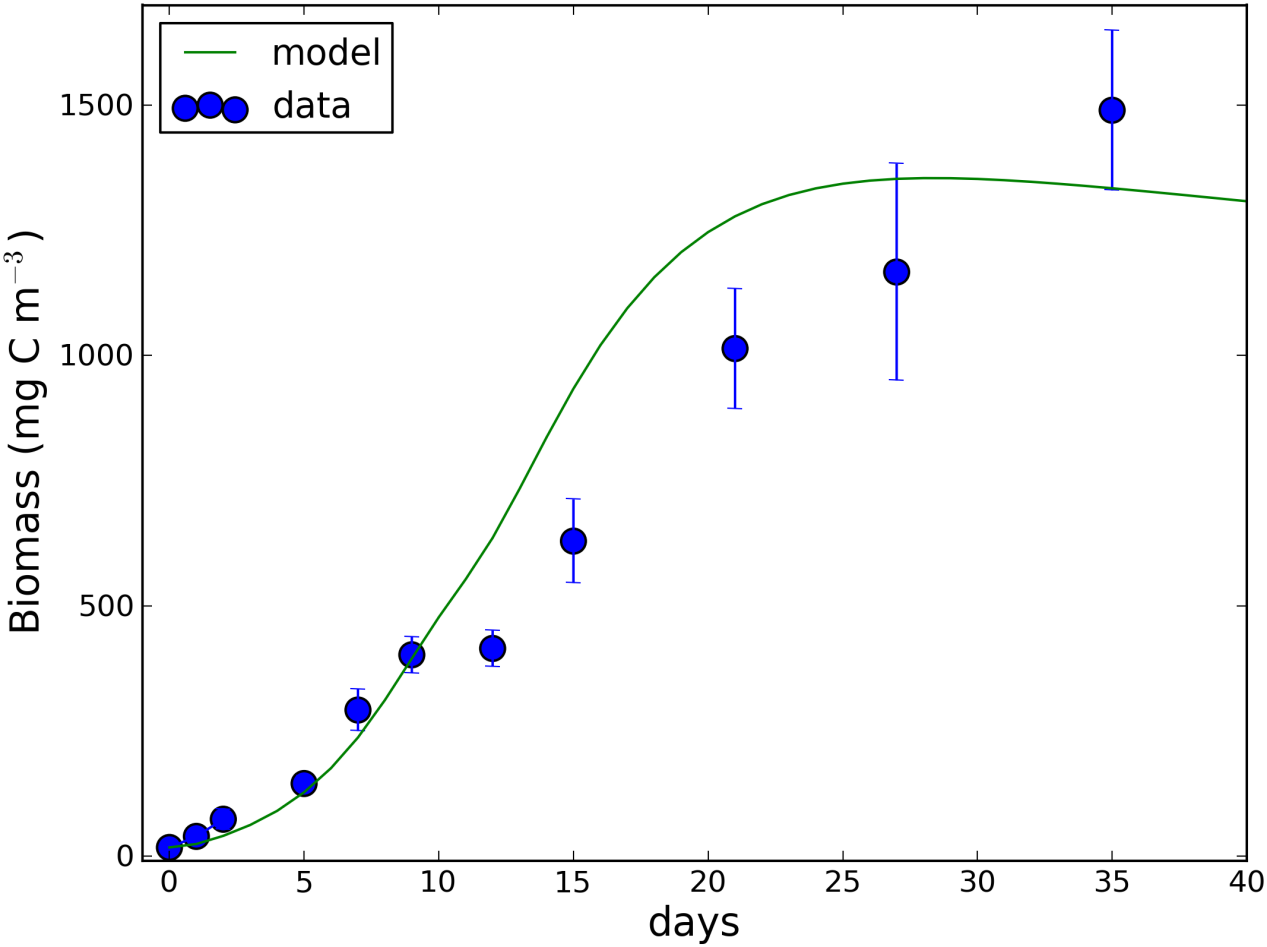
Observed and simulated bacteria biomass.

Cellular toxin and toxin to carbon ratio are displayed in panels G and H, respectively. The toxin concentration (spanning from 1 to 70 mg C m^-3^) seemed to follow the temporal evolution of carbon (panel A) and is in good agreement with the observations. The simulated toxin to carbon ratio increased steadily until day 27, consistently with the observed values. The increase of the toxin to carbon ratio was slow in the first days of the culture and it became steeper after day 10, concomitantly with the observed decrease in chlorophyll and chlorophyll to carbon ratio and the increase in carbon to nutrient ratios. The model simulated a toxin to carbon ratio ranging from 0.5 to 1 (μg C mg C^-1^) in correspondence of C:P and C:N ratios ranging from 100 to 150 and 8 to 6 (mol mol^-1^), respectively. Simulated toxin to carbon ratio increased up to 2.8 (μg C mg C^-1^) concomitantly with a C:P and C:N ratios ranging from 180 to 270 and 7 to 12 respectively. Simulated bacteria (Fig 4) were very close to the observations in the first 15 days of the culture. From day 15 to day 30 the model simulated a maximum of biomass (~1350 mg C m^-3^ at day 28) and then a (slowly) declining phase. To the contrary, observed bacteria were monotonically increasing even at the end of the culture.

### Sensitivity analysis with respect to processes

**Fig 5:**
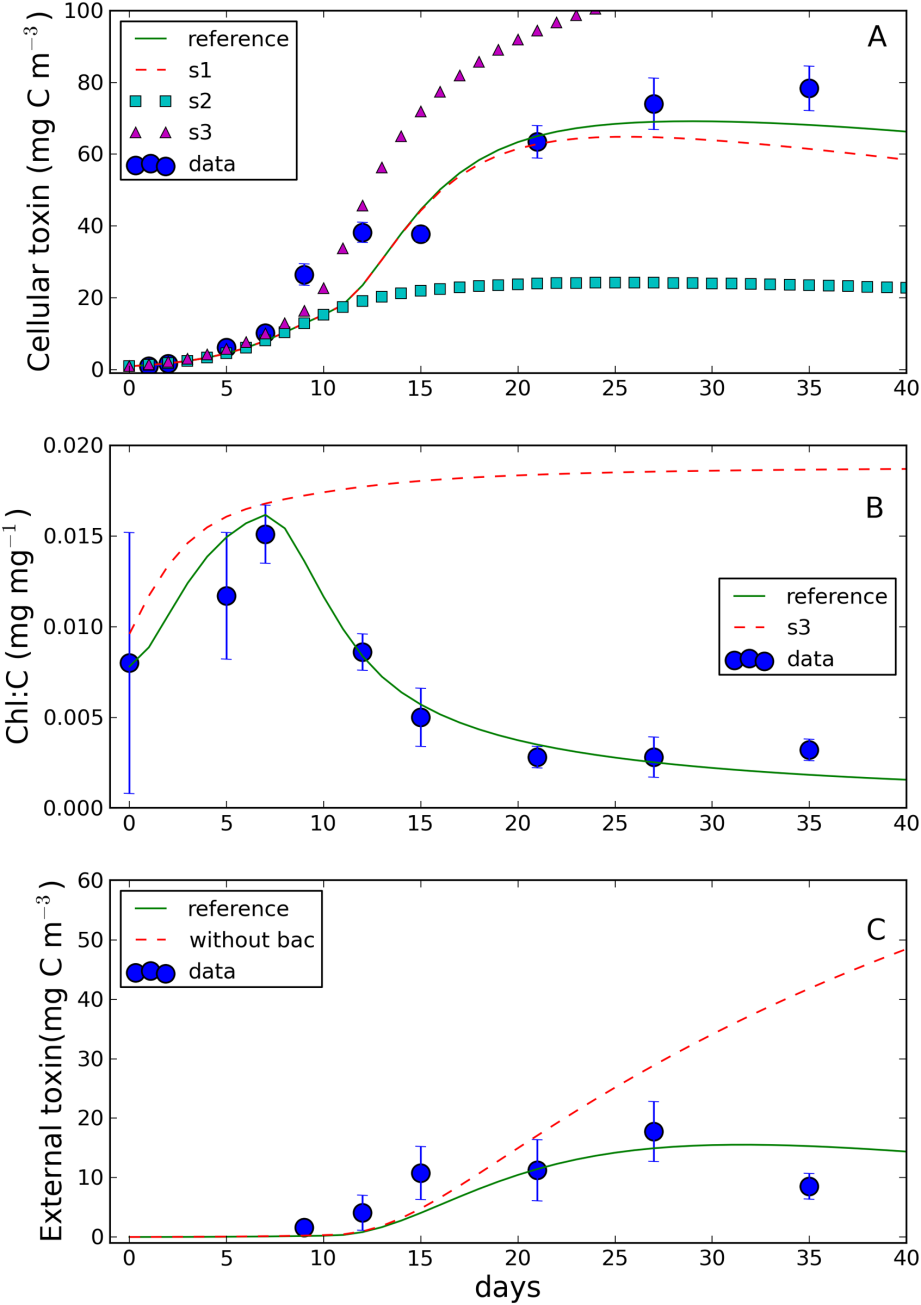
Sensitivity analysis with respect to selected model processes (experiment S1-3, see the text for explanation). (A) simulated and observed intracellular toxin; (B) modelled and observed chlorophyll to carbon ratio (Chl:C). (C) external toxin concentrations simulated with and without bacteria (bac). Data (with standard deviation) are also displayed.

**Fig 6:**
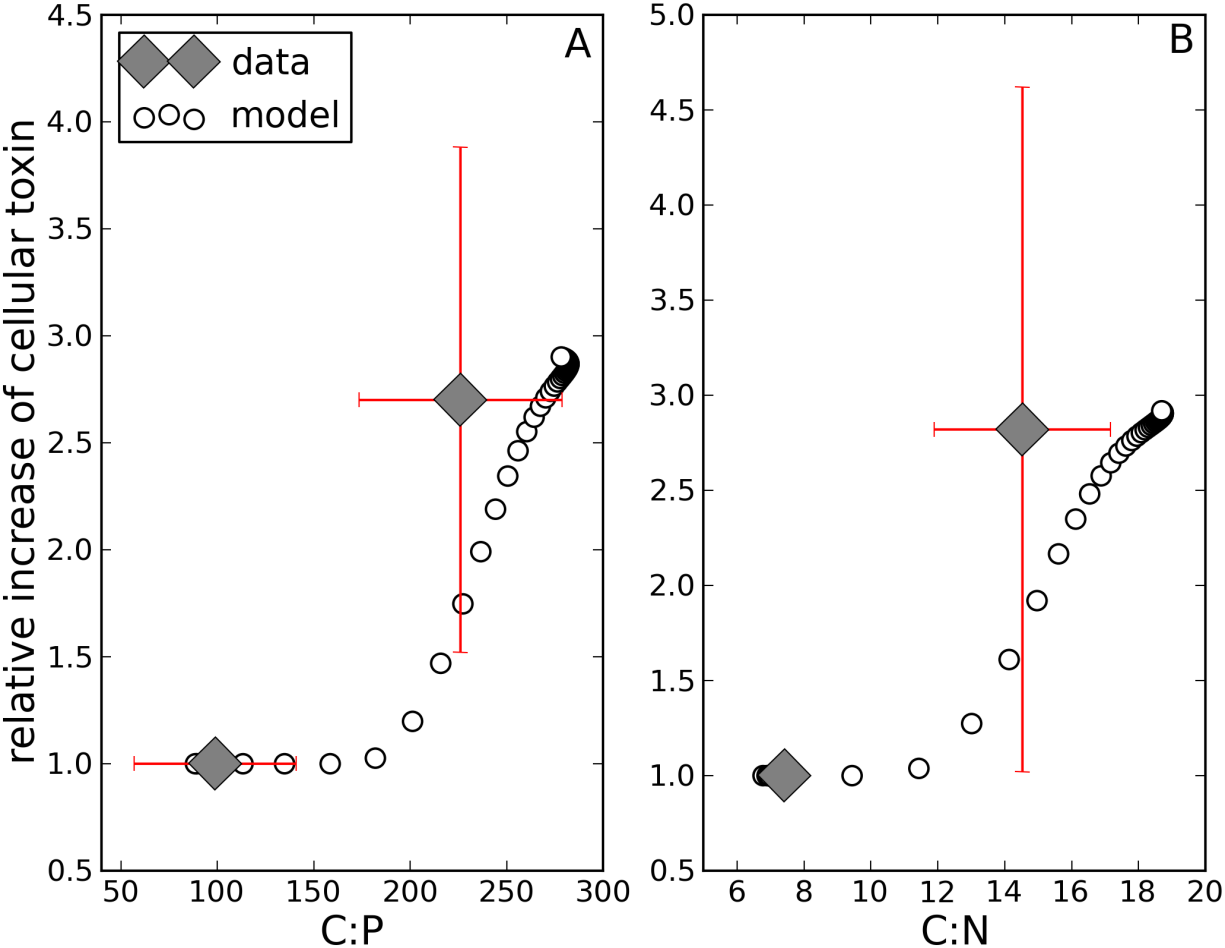
Simulated and observed changes in toxin content in response to C:P (A) and C:N (B) cellular ratios. Simulated toxin to carbon ratios have been normalized by the value of 0.001 (see Table 1 and Eq 1). Diamonds refer to the mean increase in cellular toxin observed in *Karenia brevis* grown under P-limited ([45], panel A) and N-limited ([44], panel B) conditions. The error bars represent the standard deviations.

The results of the sensitivity simulations S1, S2 and S3 (Fig 5) showed that all the investigated processes significantly improved the simulations, taking the model output closer to the observed values. As expected, the reduction in basal respiration (S1) affected only the simulation of the last few days of the experiment, when the nutrient stress was high. Conversely, the nutrient dependent reduction in chlorophyll synthesis (S2) and the nutrient-dependent enhancement of toxin production (S3) affected the simulation since the very beginning of the experiment. The effect of nutrients on chlorophyll synthesis, which was assumed to begin at a relatively high nutrient to carbon internal ratio (see Table 1 and Fig 6), was also particularly important for the realistic simulation of the chlorophyll to carbon ratio, as evident in Fig 5B. The simulation without bacteria component produced identical results with respect to the simulation with bacteria with the only exception of the external toxin (Fig 5C). Without bacteria, the model predicted an accumulation of external toxin (reaching 40 mg C m^-3^ at day 35) which was not observed in the experimental data.

### Sensitivity analysis with respect to parameters

The Monte-Carlo based sensitivity analysis demonstrated that the parameter describing the maximum toxin threshold (*Q*_*Tmax*_) was the most important in the simulation of our target model variable, i.e. the maximum value of the internal toxin concentrations (see Table 2). This parameter scored the highest value of the standardized coefficient in the regression analysis of the Monte Carlo simulations. Model parameters describing algal physiology (e.g. *S*_*P*_, *α*, *Q*_*P min*_) and the parameter regulating the amount of semi-labile (light-absorbing) organic carbon in the medium (*r*_*detr*_) were important in simulating the toxin evolution and statistically significant (p<0.05). Model parameters included in the analysis (see Table 1) but not listed in Table 2 did not influence noticeably the toxin concentration, since their regression coefficients were close to zero and were not statistically significant. Importantly, the ranking provided by the sensitivity analysis was trustworthy, since the linear regression explained most of the model output variability (R^2^ = 0.91), and it was highly significant (F-statistic = 224, p<0.01).

**Table 2:** Rank of the importance of the model parameters for the target output, i.e. the maximum internal toxin concentration. The rank is based on the absolute values of the standardized regression coefficients (|β|). The list includes a subset of the model parameters in Table 1, i.e. those that have statistically significant regression coefficients (t-statistic, p< 0.05).

	Parameter	Rank	| β |
*Q*_*Tmax*_	Maximum tox:C ratio	1	0.765
*S*_*P*_	P-stress threshold	2	0.324
*r*_*detr*_	Fraction of labile DOM derived from lysis and exudation	3	0.313
*α*	Initial slope of PI-curve	4	0.206
*Q*_*Pmin*_	Minimum P/C ratio	5	0.187
*r*_*A*.*res*_	Respired fraction of PP	6	0.111
$s_{P}^{res}$	P-stress threshold for respiration decrease	7	0.097
*r*_*B*.*res*_	Basal respiration rate	8	0.088
*Q*_*10*_	Q_10_ parameter	9	0.073
*p*_*A*.*exu*_	Fraction of excreted carbon	10	0.069
*r*_*lys*_	Minimum lysis rate	11	0.063
*φ*	Carbon fraction invested in toxin production under nutrient stress	12	0.055
$Q_{N\;min}^{chl}$	Minimum N/C ratio for chlorophyll synthesis	13	0.049
$s_{N}^{upt}$	N-threshold for max. uptake	14	0.033
*S*_*N*_	N-stress threshold	15	0.032
*σ*_*P*_	Extinction coefficient of algal biomass	16	0.0284
*Q*_*Tmin*_	Carbon fraction invested in basal toxin production	17	0.0283
$s_{N}^{chl}$	N-stress threshold for chlorophyll synthesis	18	0.023

### Comparison with literature data

The simulated relative increase of the toxin to carbon ratio (tox:C) as function of internal C:P and C:N ratios is displayed in Fig 6 along with values related to the dinoflagellate *Karenia brevis* estimated from Hardison et al. [44,45]. Model tox:C remained constant until the carbon to nutrient ratio reached a value of 170 and 12 (C:P and C:N, respectively). When these threshold values were reached, tox:C increased up to three times with respect to the values simulated under lower C:P and C:N ratios. The relative increase of tox:C observed in *K*. *brevis* grown under nutrient depletion conditions reached an average value of 2.6 under P-limitation and 2.8 under N-limitation. However, the variability among the different strains considered was remarkable, as indicated by the standard deviation bars shown in Fig 6. The model predicted an increase in tox:C (starting when C:P > ~170) which was consistent with the increase in tox:C observed in *K*. *brevis* under P-limitation. The agreement between simulated and observed tox:C increase for N-limited condition was less satisfactory since the model predicted a slower increase of tox:C with respect to C:N. The model also simulated C:N values higher than the observed one. It should be stressed, however, that the comparison under N-limitation condition is biased by the reduced number of data used (only two experiments were available).

## Discussion

**Fig 7:**
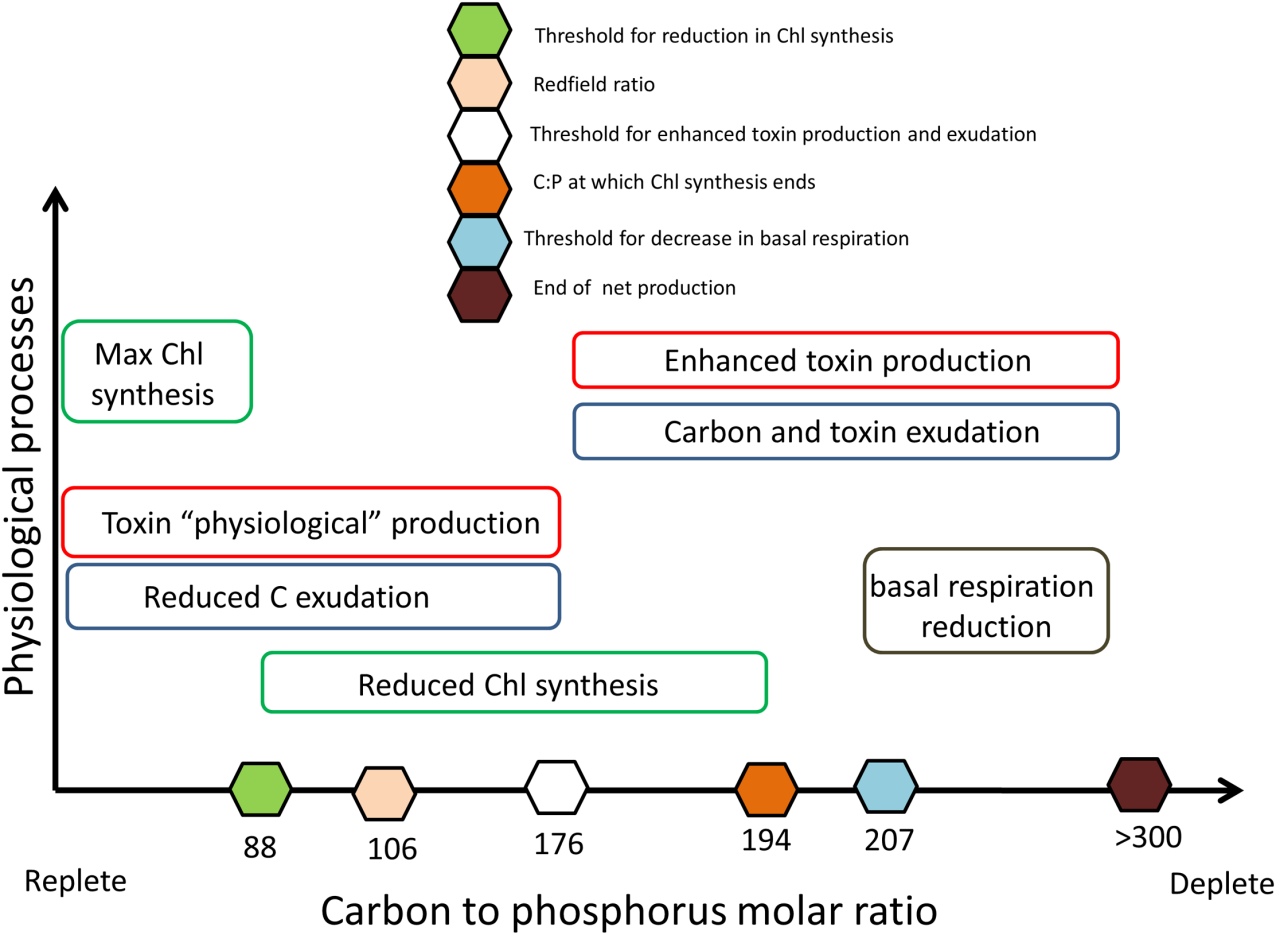
Conceptual model linking algal nutritional status (x-axis) to key physiological processes (y-axis). The sequence of processes triggered by increasing values of the carbon to phosphorus molar ratio, i.e. by shifting from high to low nutrient regimes, can be followed from the left to the right in the scheme. This is also the direction of the temporal evolution of the culture experiment, where nutrients were progressively depleted by the algae.

Our modelling analysis provided a conceptual framework describing the feedbacks between nutrient availability and cellular physiology of dinoflagellates species producing C-rich toxins (Fig 7). Notably, our approach described the nutritional cellular stress of the algae by using internal nutrient quota (i.e. carbon to nutrient ratio) rather than the nutrient ratio in the medium and this allowed us to represent cellular stress in a more realistic way [46].

### Modelling *O*. cf. *ovata* physiology

Our model was constructed by analyzing the behavior of the dinoflagellate *O*. cf. *ovata*, a species representing a new and less known issue compared with other toxic microalgae. The toxins produced by this organism are characterized by a very high molecular weight, high C:N ratio (about 43), and their production is likely to represent a high cost in carbon and energy terms. Data were obtained from *O*. cf. *ovata* cultures performed in standard nutrient conditions and followed to a very late stationary phase. This allowed us to investigate the evolution from a nutrient replete to a nutrient limitation state.

Under high nutrient regimes (as simulated in the first few days of culture) *O*. cf. *ovata* was able to actively grow, synthesizing the different cellular constituents including toxins. During the first 7 days of culture, the algae reached the maximum chlorophyll cellular content and the “physiological” level of toxins. Both chlorophyll and toxin to carbon ratios would stay approximately constant if the condition of light and nutrients were constant (balanced growth). This has already been observed for chlorophyll in cells growing under constant nutrient and light conditions [37,47]. Polysaccharide excretion was kept at a minimum physiological rate (here assumed to be 20% of the gross carbon fixation), and the toxins were not released into the environment. Under these conditions, we hypothesized that *O*. cf. *ovata* was performing a phototrophic metabolism while the cellular toxin may act as carbon (and nitrogen) storage.

As soon as the internal nutrient concentration started to decrease (day 7, Fig 3D and 3E), a sequence of physiological adjustments took place. Interestingly, chlorophyll synthesis was the first process undergoing a strong reduction. The model suggested that the carbon invested in photosynthetic compounds started to decrease when the internal nutrient level was higher than the Redfield ratio (Figs 3 and 5). However, when both chlorophyll and chlorophyll to carbon ratio started to dramatically decrease (from day 7, Fig 3), the biomass was still exponentially growing, suggesting that *O*. cf. *ovata* was able to maximize photosynthetic efficiency even with a reduced light harvesting apparatus.

The reduction in chlorophyll content per cell represents a common response of photosynthetic cells to nitrogen limitation; however, when microalgae are grown under replete conditions, chlorophyll content patterns vary from being stable for days after external nutrients were depleted [48] to decreasing concurrently with external and internal nutrient decrease [49,50]. As reported in other dinoflagellates, the observed chlorophyll to carbon ratio values in *O*. cf. *ovata* decreased as soon as the C:N ratio started to increase and reached values which are among the lowest reported [51]. This result could be due to a mixotrophyc metabolism which allows changes in trophic behavior (in this case driven by cell nutrient state) shifting from a prevailing phototropic to a prevailing heterotrophic mode for nutrient supplies. Indeed under inorganic nutrient deficiency mixotrophs would strategically survive and grow getting organic N and P by osmotrophy and/or phagotrophy [52,53] and thus, from this perspective, chlorophyll biosynthesis could become a negative energetic cost-benefit investment as suggested by Raven [54].

In the investigated system, the contribution of the dissolved organic matter to *O*. cf. *ovata* nutrition could not be assessed. However, the ability of this species to utilize organic matter is testified by the enhancement of alkaline phosphatase activity under P-limitation recently reported [55]. Moreover, the absence of prey items of a suitable size range in the system did not allow us to test the contribution of nutrients’ supply from phagotrophy. Indeed, while micropredation has been proposed for this alga [56] and observed for other *Ostreopsis* species preying on naked ciliates [57], predation on bacterial cells would require feeding strategies [53] not yet assessed for *Ostreopsidaceae* species.

Bacteria could also impact algal dynamics by making nutrients available to the algae, according to their more traditional role as remineralizers or by competing for nutrients. However, the model simulation run without the bacteria component did not change the results shown in Fig 3, with the exception of the external toxin concentration (Fig 5C), as previously hypothesized by Vanucci et al. [58]. This suggests that bacteria, while actively assimilating the toxin in the medium are not significantly impacting on algal growth and toxin production. However, given the complexity of the algal-bacteria interactions which go well beyond the competition for nutrients [59,60], additional experiments are required to better conceptualize and model the role played by bacteria on the growth and toxicity of *O*. cf. *ovata*.

With the progression of *O*. cf. *ovata* growth, the level of cellular nutrients further decreased, from day 10–11 onwards (C:P ratio >175), and the investment in toxins was enhanced concomitantly with an enhanced release of both carbon and toxins into the environment. It is interesting to note that the increased production of toxins and their external release began at a lower nutrient level with respect to the decrease in chlorophyll synthesis, suggesting that *O*. cf. *ovata* was entering into a “phase two” of its strategy to face adverse nutrient conditions. The cells increased the toxin content until approaching a critical toxin to carbon ratio represented by the parameter *Q*_*Tmax*_. Importantly, the model assumed that the enhanced production of toxins depended on the biomass standing stock and not on its production rate. This suggests that this alga is able to synthesize toxins from carbon previously photosynthesized and to produce toxins even when the environmental conditions do not allow a net growth. The observed behavior is in agreement both with the carbon to nutrient balance hypothesis (and thus with the idea that carbon surplus in plant cells is utilized to build up defense mechanisms), and with the reported observation that C-rich toxins generally increase under N and P limitation [4].

The relative importance of nitrogen or phosphorus in this species was difficult to assess because the two nutrients were depleted nearly at the same time so that in our experimental system we observed a toxin increase when both C:P and C:N were higher than the Redfield ratio.

The increase in intracellular toxin could represent a mechanism to discourage predation, as already proposed [46,61,62]. In this way, *O*. cf. *ovata* could limit top down control when the environment is strongly limiting because of the low level of nutrients, i.e. when it is bottom up controlled. This strategy could allow the algae to keep a sizable background concentration from which it is possible to re-build a healthy population when the environmental conditions revert back to positive. The extrusion of polysaccharide matrices and toxins along with the decrease in cellular nutrient content (accumulation of carbon) are all factors potentially acting as deterrents for grazers [5,20,25,63,64].

When the cellular nutrient to carbon ratio further decreased (C:P >207 in Fig 5), a third process entered into play. In the last days of the culture, C:P and C:N intracellular molar ratios reached extreme values, being very far from the values of 106 and 6.6 proposed by Redfield [17,18] as mean values for marine microalgae. In order to reproduce such low nutrient values we had to assume reduced metabolic losses (basal respiration and mortality rates). In particular, a progressive (reversible) reduction in basal respiration rate, starting from a carbon to phosphorus ratio of ~200 was necessary for the model to simulate the high concentration of algal biomass and toxins observed at the end of the culture (Fig 4). The need to include this process supports the hypothesis that *O*. cf. *ovata* forms resting stages at extremely limiting nutrient conditions [65,66].

The sensitivity analysis (Table 2) identified the most important parameters for the simulation of the maximum value of toxin. The analysis highlighted that the parameter regulating the maximum amount of cellular toxin (*Q*_*Tmax*_) should be estimated with great care to adequately simulate the intracellular toxin concentration. Notably, the analysis revealed that the parameters regulating the physiology of the alga (e.g. photosynthetic parameters, parameters regulating internal nutrient level, loss terms etc.) are also very important. This information is valuable to properly calibrate the model when, in future works, we will apply it in realistic marine ecosystem simulations. Finally, we noted that most of the new parameters introduced into the model ranked relatively low in the sensitivity analysis. This suggests that the good agreement between data and simulations was not due to an over-parameterization of the model, but to the inclusion of key processes in the model structure.

### Model validation and applicability

In order to assess to what extent the proposed model could be applicable to other species producing C-rich toxin, we have compared model simulations with the data reported in the literature for *Karenia brevis*, a dinoflagellate producing brevetoxin (classified as C-rich toxin [4]). Although the absolute value of the C-toxin as a percentage of the total carbon was much higher in *K*. *brevis* (up to 5%) than in *O*. cf. *ovata* (<1%), the trend and the value of the relative increase of the tox:C ratio as function of carbon to nutrient ratios simulated by the model was in good agreement with the observations (Fig 6). We have used the dataset reported in Hardison et al. [44,45] as it was among the few including measurements of toxin to carbon ratios and carbon to nutrient ratios which are required for a quantitative model comparison. However, from a more qualitative point of view, our model is in agreement with other observations regarding C-rich toxin producing dinoflagellates. For example, Adolf et al. [67], studying toxin production in 6 different strains of *Karlodinium veneficum* (producing the C-rich karlotoxin), observed high values of cellular toxin concentration (up to 21 pg of toxin per cell) under high C:P values (up to 470). These values when normalized by the values corresponding to more balanced C:P (i.e. C:P closer to the Redfield ratio) gave a relative increase ranging from 2.7 to 10.7. Johansson et al. [68] observed an increase in okadaic acid production in *Dynophysis acuminata* when cultured under N and P-limiting conditions. More specifically the mass of okadaic acid per cell with respect to the nutrient replete culture increased by a factor 4.3 and 20 for P-limited and N-limited conditions, respectively. Johansson and Granéli [69], when studying *Chrisochromulina polylepis*, observed an increase of a factor 2 in the algal toxicity (given as hemolytic activity), concomitantly with a variation of the C:P ratio from 111 to 249.

The absolute values of internal toxins (and the associated toxicity) are regulated in the model by the parameters *Q*_*Tmax*_ and *Q*_*Tmin*_. This parameters are highly variable among microalgae and even within different strains of the same species [44,45,70,71,72]. For this reason, in order to assess the model capacity to simulate a general behavior, we have normalized the toxin quota observed (and modelled) under high carbon to nutrient ratio by the values observed (and modelled) under balanced growth. Results shown in Fig 6 and the broad comparison with the literature highlighted that the model is able to reproduce a behavior widely observed among toxic dinoflagellates. This means that the model can be used to simulate the stoichiometric regulation of C-rich toxins in different dinoflagellates when species-specific parameters (i.e. *Q*_*Tmin*_ and *Q*_*Tmax*_) are properly tuned.

Finally, we recall that our model only describes the relationship between nutrients (throughout the cellular nutritional status) and toxin production, and it does not account for other biotic processes, such as grazing, which can also stimulate toxin production in dinoflagellates [73,74]. These factors are important to fully understand the environmental regulation of toxin production and should be considered in future model developments meant to simulate algal toxicity in a realistic ecosystem framework.

## Conclusions

We have developed a numerical model by interpreting experimental data describing growth and toxin production in *O*. cf. *ovata*. By analyzing model simulations and comparing them with experimental data, we have provided a conceptual framework linking the nutritional status to toxin production in this alga. In particular, we have identified four physiological processes directly associated with an increasing state of intra-cellular nutrient stress (increase of C:P and C:N cellular ratio): i) a sharp decrease in chlorophyll synthesis ii) an enhancement of toxin production, iii) the release (exudation) of toxin in the environment and iv) a reduction in metabolic maintenance leading to the formation of resting stages.

By comparing model simulations with literature data, we have highlighted that our model has the potential to simulate the stoichiometric regulation of C-rich toxins in different marine dinoflagellates. The next step will be to implement the presented formulation in a comprehensive ecosystem modeling framework (for example the ERSEM-GOTM system [21]) and to use it to investigate and predict the effect of nutrient supply in the formation and evolution of harmful algal blooms.

## Supporting Information

S1 Fig Relationship between call carbon and cell volume. (TIF)

S1 File Appendix A (Model equations). (DOCX)

S2 File Dataset (Experimental data). (XLSX)

S3 File Dataset (Model simulations). (XLSX)

S4 File Dataset (Model simulations). (XLSX)

S5 File Dataset (Model simulations). (XLSX)
