# Supplementary figures and images for: Modelling the Stoichiometric Regulation of C-Rich Toxins in Marine Dinoflagellates
Source: PLoS One. 2015 Sep 23;10(9):e0139046. doi: 10.1371/journal.pone.0139046 (PMC4580455; doi:10.1371/journal.pone.0139046)

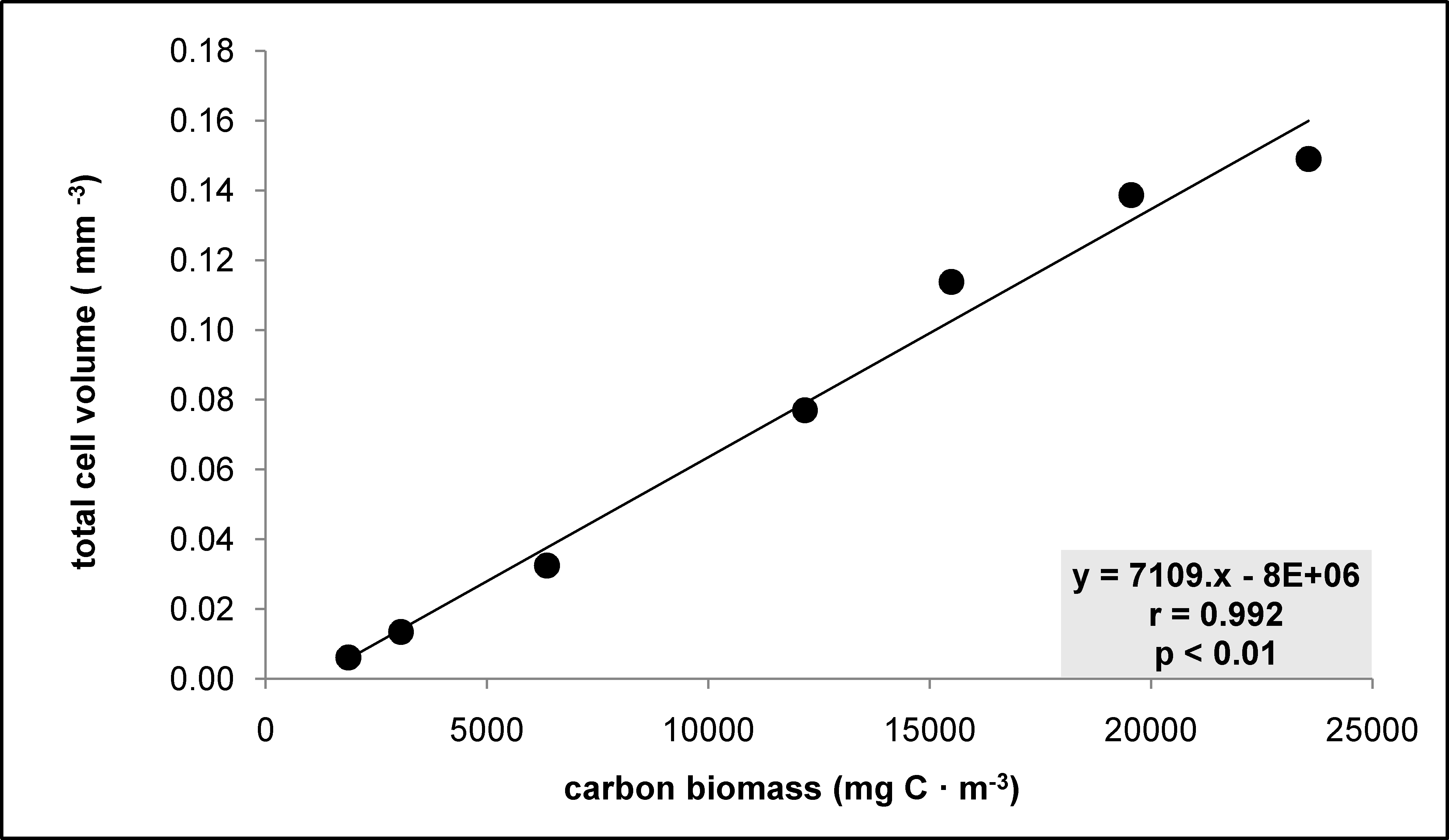

Supplement: S1 Fig — (TIF) [file pone.0139046.s001.tif]
