# Supplementary material for: Modelling the Stoichiometric Regulation of C-Rich Toxins in Marine Dinoflagellates
Source: PLoS One. 2015 Sep 23;10(9):e0139046. doi: 10.1371/journal.pone.0139046 (PMC4580455; doi:10.1371/journal.pone.0139046)
Supplement: S1 File — (DOCX) [file pone.0139046.s002.docx]

**Appendix A**

Model equations are given in the following sections. Model parameters are listed in Table I.

**Phytoplankton processes**

Light extinction $\sigma$(m^-1^) is calculated as a function of water, particulates and dissolved semi-labile organic carbon present in the medium:

$\sigma= \sigma_{bg}+ \left( \sigma_{P}\cdot P^{C} \right)+\left( \sigma_{sl}\cdot R_{sl}^{C} \right)+\left( \sigma_{POM}\cdot R_{POM}^{C} \right)$ *(1a)*

where $P^{C}$, $R_{sl}^{C}$ and $R_{POM}^{C}$ are the carbon concentration of algal biomass, semi-labile DOC and POC, respectively.

The average light ($L^{av}$) to which algal biomass is exposed to is calculated as follows:

$L^{av}=\frac{L}{\sigma\cdot\Delta z}\left( 1-e^{\left( -\sigma\cdot\Delta z \right)} \right)$ *(2a)*

where $L$ is the environmental Photosynthetically Active Radiation (PAR) and $\Delta z$ is the thickness of the water layer, here assumed to be 0.15 m.

The temperature response factor for phytoplankton ( $ʄ^{T}$ ) is calculated with a specific $Q_{10}$ function at any temperature value *T*:

$ʄ^{T}= {Q_{10}}^{\left[ \left( T-10 \right)/10 \right]}-{Q_{10}}^{\left[ \left( T-32 \right)/3 \right]}$ *(3a)*

The general equation for algal biomass (P) is the following:

$\frac{d P}{d t}= PHOTOSYNTHESIS-RESPIRATION-LYSIS-EXUDATION$ *(4a)*

where:

$PHOTOSYNTHESIS=r_{ass}\cdotʄ^{T}\cdot\varphi\cdot P^{C}$ *(5a)*

$\varphi$ is a function describing the light limitation in phytoplankton and is given by:

$\varphi=\left\{ 1-e^{\left[ -\alpha\cdot I\cdot\theta/\left( r_{ass}\cdotʄ^{T} \right) \right]} \right\}\cdot e^{\left[ -\beta\cdot I\cdot\theta/\left( r_{ass}\cdotʄ^{T} \right) \right]}$ *(6a)*

where *I* is the PAR and $\theta$ the actual chlorophyll to carbon ratio.

The proportion of net photosynthesis directed to chlorophyll synthesis ( $\rho$ ) is given by:

$\rho=\left( \theta_{max}\cdot r_{ass}\cdotʄ^{T}\cdot\varphi\right)/\left( \alpha\cdot I\cdot\theta\right)$ *(7a)*

Algal mortality due to lysis is calculated as:

$LYSIS=r_{lys}\cdot P^{C}$ *(8a)*

Cellular carbon exudation is described by the sum of two distinct terms, activity exudation ($A.EXU$) and nutrient stress-induced exudation ($S.EXU$):

$A.EXU=PHOTOSYNTHESIS\cdot p_{A.exu}$ *(9a)*

$S.EXU=PHOTOSYNTHESIS\cdot\left( 1-NS \right)\cdot\left( 1-p_{A.exu} \right)$ *(10a)*

DOC derived by lysis and exudation is split into labile and semi-labile components using the parameter $r_{\mathrm{detr}}$ in Table I.

Algal respiration is composed by a basal metabolism (*B.RES* see eq. 10) and an activity respiration term (*A.RES*):

$RESPIRATION=B.RES+A.RES$ *(11a)*

where:

$A.RES=r_{A.res}\cdot\left( PHOTOSYNTHESIS-EXUDATION \right)$ *(12a)*

Chlorophyll loss terms due to rest respiration, exudation and lysis are modeled as for carbon.

Nutrient losses due to lysis are channeled to the dissolved organic nitrogen (DON) and phosphorus (DOP) pool, according to the phytoplankton nutrient to carbon ratio.

Phosphorus (${PO}_{4}$) uptake is given by:

${UPTAKE}_{P}=MIN\left[ {UPTAKE}_{P.req} , \left( a_{P}\cdot{PO}_{4}\cdot P^{C} \right) \right]$ *(13a)*

${UPTAKE}_{P.req}$ is composed of two terms. The first is proportional to the net photosynthesis while the second describes cellular nutrient accumulation up to a threshold value (luxury uptake):

${UPTAKE}_{P.req}=(PHOTO.-EXU.-A.RES)\cdot Q_{Pmax}+\left( Q_{Pmax}\cdot P^{C} \right)-P^{P}$ *(14a)*

where $Q_{Pmax}$ is the maximum phosphorus to carbon cellular ratio and $P^{P}$ is the cellular phosphorus content.

Nitrogen uptake takes into account of both ammonium (${NH}_{4}$) and nitrate (${NO}_{3}$):

${UPTAKE}_{N}=MIN\left[ {UPTAKE}_{N.req} , \left( a_{NO3}\cdot{NO}_{3}\cdot P^{C} \right)+\left( a_{NH4}\cdot{NH}_{4}\cdot P^{C} \right) \right]$ *(15a)*

${UPTAKE}_{N.req}=\left( PRODUCTIVITY\cdot Q_{Nmax} \right)+\left( Q_{Nmax}\cdot P^{C} \right)-P^{N}$ *(16a)*

where $Q_{Nmax}$ is the maximum nitrogen to carbon cellular ratio and $P^{N}$ is the nitrogen cellular content.

**Bacteria processes**

The temperature response factor for bacteria ($ʄ_{B}^{T}$) is calculated with a specific $Q_{10}$ function:

$ʄ_{B}^{T}= {Q_{10B}}^{\left[ \left( T-10 \right)/10 \right]}-{Q_{10B}}^{\left[ \left( T-32 \right)/3 \right]}$ *(17a)*

The general biomass equation is given by:

$\frac{d B}{d t}= {UPTAKE}_{B}-{RESPIRATION}_{B}-MORTALITY$ *(18a)*

where DOC uptake is given by:

${UPTAKE}_{B}=MIN\left[ \left( r_{ass}^{B}\cdotʄ_{B}^{T}\cdot{eO}_{2}\cdot{NS}^{B}\cdot B^{C} \right) , DOC \right]$ *(19a)*

where $B^{C}$ is the bacterial carbon biomass. ${eO}_{2}$is given by:

${eO}_{2}=O_{rel}/\left( O_{rel}+h_{ox} \right)$ *(20a)*

where $O_{rel}$ is the relative oxygen saturation and $h_{ox}$ is the oxygen concentration at which the limiting factor equals 0.5. ${NS}^{B}$is given by the expression:

${NS}^{B}=MIN\left[ \frac{{NH}_{4}+R_{DOM}^{N}}{{NH}_{4}+R_{DOM}^{N}+h_{N}} , \frac{{PO}_{4}+R_{DOM}^{P}}{{PO}_{4}+R_{DOM}^{P}+h_{P}} \right]$ *(21a)*

where $R_{DOM}^{N,P}$ are the nitrogen and phosphorus contents of DOM and $h_{N,P}$ are the nitrogen and phosphorus concentrations at which the limiting factor equals 0.5.

Respiration is composed of an activity (dependent on DOM uptake) and a basal (depending on biomass) term:

${RESPIRATION}_{B}={UPTAKE}_{B}\cdot\left[ r_{A.res}^{B}\cdot O_{rel}+r_{resOX}^{B}\cdot\left( 1-O_{rel} \right) \right]+\left( r_{B.res}^{B}\cdotʄ_{B}^{T}\cdot B^{C} \right)$ *(22a)*

where $r_{A.res}^{B}$ is the respired fraction of uptake.

Mortality is given by:

$MORTALITY=r_{lys}^{B}\cdotʄ_{B}^{T}\cdot B^{C}$ *(23a)*

DOC derived by mortality process is split into labile and semi-labile components using the parameter $r_{\mathrm{detr}}$ in Table I. Nitrogen and phosphorous cellular content are channeled into the DON and DOP pool, respectively.

Nutrient excretion is only active when nutrient ($i$ = N or P) are in excess and is given by:

${LOSS}_{i}=\left( Q_{i}^{B}-Q_{i max}^{B} \right)\cdot B^{C}$ *(24a)*

where $Q_{i}^{B}$ is the actual nutrient to carbon ratio. In case of internal nutrient shortage, eq. *23a* is replaced by the following:

${UPTAKE}_{BAC}^{i}=\left( Q_{i}^{B}-Q_{i max}^{B} \right)\cdot B^{C}\cdot\frac{i}{\left( i+h_{i} \right)}$ *(25a)*

The breakdown of semi-labile ($R_{slDOM}^{C}$) to labile DOC ($R_{lDOM}^{C}$) is described by:

$R_{lDOM}^{C}= r_{dis}\cdot R_{slDOM}^{C}$ *(26a)*
